# *In Vivo* PET Imaging of Adenosine 2A Receptors in Neuroinflammatory and Neurodegenerative Disease

**DOI:** 10.1155/2017/6975841

**Published:** 2017-11-20

**Authors:** Anna Vuorimaa, Eero Rissanen, Laura Airas

**Affiliations:** ^1^Turku PET Centre, Turku University Hospital and University of Turku, Turku, Finland; ^2^Division of Clinical Neurosciences, Turku University Hospital and University of Turku, Turku, Finland

## Abstract

Adenosine receptors are G-protein coupled P1 purinergic receptors that are broadly expressed in the peripheral immune system, vasculature, and the central nervous system (CNS). Within the immune system, adenosine 2A (A_2A_) receptor-mediated signaling exerts a suppressive effect on ongoing inflammation. In healthy CNS, A_2A_ receptors are expressed mainly within the neurons of the basal ganglia. Alterations in A_2A_ receptor function and expression have been noted in movement disorders, and in Parkinson's disease pharmacological A_2A_ receptor antagonism leads to diminished motor symptoms. Although A_2A_ receptors are expressed only at a low level in the healthy CNS outside striatum, pathological challenge or inflammation has been shown to lead to upregulation of A_2A_ receptors in extrastriatal CNS tissue, and this has been successfully quantitated using* in vivo* positron emission tomography (PET) imaging and A_2A_ receptor-binding radioligands. Several radioligands for PET imaging of A_2A_ receptors have been developed in recent years, and A_2A_ receptor-targeting PET imaging may thus provide a potential additional tool to evaluate various aspects of neuroinflammation* in vivo*. This review article provides a brief overview of A_2A_ receptors in healthy brain and in a selection of most important neurological diseases and describes the recent advances in A_2A_ receptor-targeting PET imaging studies.

## 1. Introduction

Adenosine is a highly bioactive molecule, which is stored inside cells as adenosine triphosphate (ATP) and transported to the extracellular space by transporter molecules or catabolized into adenosine extracellularly by ectoenzymes CD39 and CD73 [1, 2]. It is rapidly transported back into cells and degraded into inosine or phosphorylated back to adenosine monophosphate (AMP) by adenosine deaminase and adenosine kinase, respectively [1]. Within the central nervous system (CNS), neurons and glia release adenosine, and concentration of adenosine increases in the extracellular space following ATP release during inflammation or cellular trauma [3]. Adenosine is ubiquitous, but short-lived [4]. It confers its biological effects locally via four adenosine-binding purinergic P1 receptors: A_1_, A_2A_, A_2B_, and A_3_ [1]. This leads to physiological regulation of a variety of important CNS functions, such as modulation of neuronal excitability, release and uptake of neurotransmitters, and modification of synaptic plasticity [5–9]. In addition, adenosine receptors have a vasoactive function [10] and an important role in controlling inflammatory events [11]. In particular, signaling through the adenosine 2A (A_2A_) receptor has been described as a potent regulator of inflammation [12]. In healthy CNS, A_2A_ receptor expression is the greatest in the neurons of the basal ganglia, where it is involved in motor control in conjunction with dopamine 2 (D_2_) receptors, but under pathological conditions, A_2A_ receptor expression has been demonstrated also in brain areas outside the striatum [13]. Importantly, pharmacological targeting of A_2A_ receptors using antagonists or agonists may have important therapeutic implications in several CNS diseases [14]. A_2A_ receptor-binding radioligands have enabled* in vivo* positron emission tomography (PET) imaging of A_2A_ receptor expression. The human A_2A_ receptor PET studies have focused either on the striatal neuronal A_2A_ receptor expression, relevant to movement disorders [15, 16], or on A_2A_ receptor upregulation in the white matter in the context of neuroinflammatory disease [13]. This review will provide a brief overview of A_2A_ receptors in healthy brain and will describe their involvement in a selection of most important neurological diseases, such as Parkinson's disease (PD), Huntington's disease (HD), stroke, and multiple sclerosis (MS). The role of* in vivo* PET imaging in advancing the understanding of the A_2A_ receptor biology within the CNS will be discussed.

## 2. A_2A_ Receptor Expression in Various CNS Compartments and Cell Types

### 2.1. Neurons

**Figure 1:**
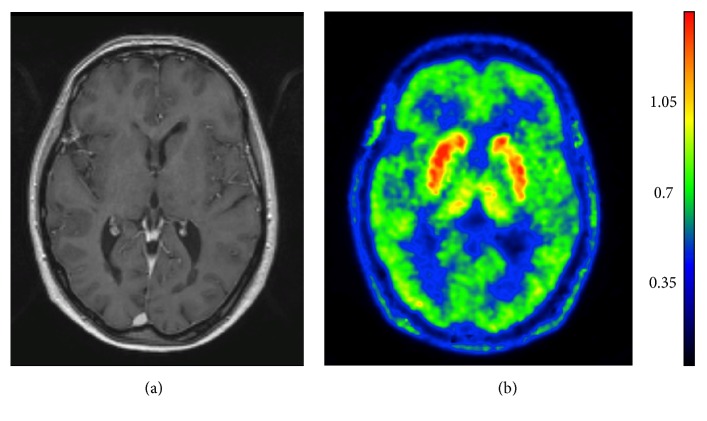
MRI and [^11^C]TMSX PET images of a healthy subject. Axial T1 gadolinium-enhanced weighted MR image (a) and corresponding parametric [^11^C]TMSX PET image (b). [^11^C]TMSX uptake is visualized as voxelwise distribution volume (*V*_*T*_) denoted by the color scale on the right. Strong binding to A_2A_ receptors is seen in the striatum, where A_2A_ receptors are expressed on striatopallidal medium spiny neurons.

Adenosine receptors are far more abundant in the brain than in any other organ [17]. In healthy brain, A_2A_ receptor expression is most prominent in neurons of the basal ganglia (Figure 1) [18, 19]. A_2A_ receptors are also expressed in neurons in the neocortex and the limbic cortex [20–22], where they are predominantly present in nerve terminals, albeit with a density 20 times lower than that found in the basal ganglia [20]. The distribution of A_2A_ receptors is similar in rodents and humans [23, 24]. However, the level of extrastriatal A_2A_ receptor expression appears to be higher in humans than in rodents [18]. In the basal ganglia, the A_2A_ receptors are colocalized with dopamine 2 receptors in the striatopallidal gamma-aminobutyric acid (GABA)ergic neurons containing enkephalin [18, 25]. A_2A_ receptors are mostly localized postsynaptically [26] but are also found presynaptically on glutamatergic nerve terminals, where they contact the direct-pathway medium spiny neurons [27] and can form heteromers with A_1_ receptors [9]. A_2A_ receptor antagonists have also been found to modify the N*-*methyl-D-aspartic acid (NMDA) receptor subunit composition in transgenic R6/2 mice [28]. The ability of A_2A_ receptors to control the release of glutamate in the cerebral cortex [8, 29, 30], hippocampus [21, 22, 31], and striatum [32–38] has led to the hypothesis that the reduction in glutamate release might be the explanation for the neuroprotective effects of A_2A_ receptor antagonism [39, 40]. The inhibition of glutamate release by A_2A_ receptor antagonism seems, however, strongly time dependent in relation to lesion formation and animal age. Quinolinic acid (QA) induced glutamate release is almost completely blocked in rat striatum by pretreatment with A_2A_ receptor antagonist SCH58261 [39] but this effect of A_2A_ receptor antagonist is reversed two weeks after QA lesion, when SCH58261 significantly increases glutamate outflow [37]. Similarly, spontaneous outflow of glutamate in response to SCH58261 treatment in young rats is different from that in aged ones [35]. Future studies are awaited to confirm the usefulness of A_2A_ receptor antagonism in protection from glutamate-related neurotoxicity in various neurodegenerative conditions.

### 2.2. Endothelial Cells

Brain endothelial cells, together with astrocytes and pericytes, form the blood-brain barrier (BBB), a physical barrier that protects the CNS against blood pathogens and prevents immune cell infiltration [41]. Endothelial cells of the BBB are linked together with occludins, claudins, and junctional adhesion molecules (JAMs) that form the tight junctions that inhibit almost all the paracellular transportation through the BBB [42]. Although the BBB allows less passing than most endothelial barriers under normal circumstances, during CNS infection, trauma or autoimmunity immune cells from the periphery gain access to the CNS parenchyma [43]. One possible mediator controlling BBB permeability is the adenosine A_2A_ receptor [44].

A_2A_ receptors are expressed on human brain endothelial cells together with adenosine-forming enzymes, CD39 and CD73 [45–47]. A_2A_ receptor expression has also been described on mouse and rat brain endothelial cells [48]. Evidence from animal studies suggests that activation of the A_2A_ receptors promotes an increase in BBB permeability to macromolecules [48]. However, another study suggested that the increased production of adenosine via induction of the adenosine-generating ectoenzyme CD73 on primary human brain endothelial cells after interferon beta (IFN-*β*) treatment leads to improved barrier function, but the target molecule of adenosine in this particular setting remains uncertain [49]. Activation of A_2A_ receptors with a broad-spectrum adenosine receptor agonist 5′-*N*-ethylcarboxamidoadenosine (NECA) or A_2A_ receptor-specific agonist Lexiscan (regadenoson, FDA approved for use as a pharmacological stress agent for radionuclide myocardial perfusion imaging) increased BBB permeability to macromolecules such as 10 kD dextrans (NECA and Lexiscan) and 70 kD dextrans (NECA) and antibodies to *β*-amyloid (NECA)* in vivo* [48]. Increase in barrier permeability after A_2A_ receptor agonist treatment was linked to changes in cell cytoskeleton structure, measured as decreased transendothelial cell electrical resistance (TEER) and actomyosin stress fiber formation, as well as decreased expression of tight junctions molecules, most strongly occludin [48]. Similar cytoskeletal changes were observed in primary human brain endothelial cells after treatment with A_2A_ receptor agonist [45]. Furthermore, A_2A_ receptor agonist treatment has been shown to promote paracellular transendothelial migration of lymphocytes through a model of human BBB [45]. In peripheral blood vessels the role of A_2A_ receptors in the control of vessel permeability remains less clear, as A_2A_ receptor agonists have been shown, depending on conditions, to either increase or decrease endothelial permeability [50–54].

### 2.3. A_2A_ Receptor in Choroid Plexus

In order for immune cells to gain access to the CNS, they need to cross either of the protective barriers between the periphery and CNS: the BBB or the blood-cerebrospinal fluid barrier (BCSFB). The BCSFB is formed by the choroid plexus and is made up of fenestrated capillaries, which are surrounded by parenchyma covered with epithelial cells that, like the BBB endothelial cells, are joined together by tight junctions [55, 56]. A_2A_ receptors are expressed on choroid plexus endothelial cells, where they seem to regulate lymphocyte migration into the CNS [57, 58]. This was also shown to contribute to the development of experimental autoimmune encephalomyelitis (EAE), the animal model of MS [57]. Here, ATP released from damaged cells within the CNS is hydrolyzed to adenosine by choroid-plexus-expressing ectoenzymes CD39 and CD73. Adenosine binds to the A_2A_ receptor and facilitates the lymphocyte entry via enhancing CX3CL1 expression at the choroid plexus [59]. Lack of A_2A_ receptors results in reduced lymphocyte entry [57].

### 2.4. A_2A_ Receptors in Glia

A role for A_2A_ receptors has been described in oligodendrocyte differentiation. A_2A_ receptor expression has been demonstrated on oligodendrocyte precursor cells [60], and A_2A_ receptor signaling seems to inhibit oligodendrocyte progenitor cell maturation, whereas A_1_ receptor signaling promotes it [61, 62]. Under chronic inflammatory or neurodegenerative conditions, A_2A_ receptor expression has been demonstrated also in other CNS areas and cell types, such as microglia [63, 64] and astrocytes [65]. In several neurodegenerative CNS diseases astrogliosis can contribute to the disease pathogenesis by contributing to cellular death. Interestingly, A_2A_ receptor antagonism might contribute to control of astrogliosis, as A_2A_ antagonists SCH58261 and KW6002 were shown to significantly inhibit signs of astrogliosis in a primary cell culture of striatal rat astrocytes [66]. Similarly, A_2A_ receptor activation led to morphological changes in cultured microglia indicative of further microglial activation, a phenomenon which could be blocked using A_2A_ receptor antagonists [63]. Hence, astrocytes and microglia might provide the central link between A_2A_ receptor-mediated effects in neuroinflammatory and neurodegenerative diseases, which will be discussed in the next chapters.

## 3. A_2A_ Receptors in Neurodegenerative Disease

### 3.1. Parkinson's Disease

A_2A_ receptors are abundantly expressed on neurons in the striatum [18, 19], where they colocalize with dopamine 2 receptors on the GABAergic striatopallidal neurons of the “indirect pathway” [25, 67]. In the classical model, direct and indirect pathways work together in fine-tuning movement by exciting and inhibiting the cerebral motor cortex, respectively. Presently, it is acknowledged that complex interplay is likely to occur between these two pathways [68]. A_2A_ and D_2_ receptors are functionally antagonistic, as A_2A_ receptor antagonist can exert a similar effect on motor control as D_2_ agonists. This effect is explained by the receptors' opposing effect on adenylyl cyclase and by their ability to form heteromers [69, 70]. In PD, loss of dopaminergic input from substantia nigra leads to unbalance of the sensitive motor behavior controlling system. Initially effective solution to depletion of dopamine in PD has been dopamine replacement therapy by levodopa. However, in chronic levodopa treatment, patients start experiencing dyskinesias and symptoms of “wearing-off”; that is, there will be motor fluctuations as the effective time of the medication shortens [71]. Because A_2A_ receptor antagonists exert suppression similar to D_2_ receptor activation on the medium spiny neurons of the indirect pathway, they have been studied as an add-on therapy to levodopa in PD [72].

PET imaging using A_2A_ receptor-binding radioligands has been used to evaluate striatal A_2A_ receptor expression in PD* in vivo*. Distribution volume ratio (DVR) of [^11^C]TMSX ([7-N-methyl-^11^C]-(E)-8-(3,4,5-trimethoxystyryl)-1,3,7-trimethylxanthine) binding in the putamen was shown to be higher in PD patients with dyskinesias (disease duration: 11.1 ± 7.2 years) compared to healthy controls [16]. On the other hand, in drug-naïve patients (disease duration: 2.0 ± 1.2 years) there was no significant difference in [^11^C]TMSX binding compared to healthy controls. [^11^C]TMSX DVR was, however, increased in the putamen in a follow-up scan after approximately a year of induction of antiparkinsonian therapy compared to the baseline scans, despite the absence of clinical dyskinesias [16]. Similarly, using another A_2A_ receptor-binding radioligand, [^11^C]SCH442416, and PET imaging, a significant increase was found in the binding potential in the putamen and the nucleus caudatus in PD patients with levodopa-induced dyskinesias (disease duration: 13.2 ± 5.6) compared to PD patients with levodopa treatment without dyskinesias (disease duration: 6.2 ± 3.4) and to healthy controls [15].

### 3.2. Huntington's Disease

Brain pathology in HD is characterized by striatal atrophy with a selective loss of medium spiny neurons [73]. Interestingly, neuropathological studies have demonstrated a marked loss of striatal A_2A_ receptors in early stages of HD [23, 74], and similar loss of A_2A_ receptors is reported in transgenic mouse models of HD [75–77]. Moreover, expression of mutant Huntingtin was shown to lead to reduced A_2A_ receptor expression in cell cultures by regulating transcription of the A_2A_ receptor gene [78]. Finally, A_2A_ receptor gene (ADORA2A) rs 5751876 genotype was shown to affect the age of onset of HD in humans [79]. In transgenic HD animal models, blockade of A_2A_ receptors rescues cognitive performance impaired by the disease [80, 81]. A_2A_ receptor agonists on the other hand have shown to reverse motor deficits [77], whereas blockade of the receptor worsens motor performance [76, 82].* In vivo *A_2A_ receptor-targeting PET imaging using [^11^C]KF18446 has been used to demonstrate reduced A_2A_ receptor expression in an animal model of HD [83]. Here, the binding potential of [11C]KF18446 was significantly decreased in the quinolinic acid-lesioned striatum. Thus,* in vivo* imaging of A_2A_ receptors in HD patients might provide insight into the pathologic changes in A_2A_ receptors in different stages of the disease. Moreover, PET imaging of A_2A_ receptors could be availed for interrogating treatment response to possible adenosine signaling targeting therapies in HD. To our knowledge, no A_2A_ receptor-targeting PET imaging has yet been performed in HD patients.

### 3.3. Alzheimer's Disease

A_2A_ receptors are upregulated in the frontal cortex and hippocampus in Alzheimer's disease (AD) [65, 84] and likewise in animal models of AD [5, 85]. In vitro, A_2A_ receptor antagonists prevent amyloid *β* (A*β*) induced neurotoxicity and synaptotoxicity [5, 86–88], whereas A_2A_ receptor agonists increase *Aβ* production [89]. In various animal models of AD, blockade or genetic deletion of A_2A_ receptors enhances memory function [5, 90–92]. A_2A_ receptor activation is in fact sufficient to disrupt memory even in healthy rats [93, 94]. On the other hand, treatment of APP/PS1 mice with A_2A_ receptor antagonist was shown to increase A*β*_42_ accumulation in cortical neurons (but not in the hippocampus) [95]. A_2A_ receptor activation specifically in the hippocampus was shown to impair memory, whereas in the nucleus accumbens it only induced locomotor activity instead [94]. Interestingly, activation of chimeric rhodopsin-A_2A_ receptor by light stimulated the cAMP-PKA pathway and increased CREB and c-Fos expression in the hippocampus but stimulated the MAPK signaling pathway in the nucleus accumbens [94]. Finally, Orr et al. showed that selective deletion of A_2A_ receptors from astrocytes enhanced memory in an AD animal model [65]. Even though A_2A_ receptor antagonism or deletion in animal models of AD mainly appears to exert neuroprotective effects, the causal relationship between adenosine signaling and amyloid deposition, as well as disease progression, remains unclear. More efficient therapies for halting or slowing down the course of the disease in AD are sorely needed, and anti-A_2A_ therapy appears as an intriguing option in this field. Before this, however, additional evidence of the role of A_2A_ receptors in AD as well as in other neurodegenerative diseases would be needed. Imaging A_2A_ receptors in different stages of the disease and in studying treatment response to novel emerging therapies would shed more light on the understanding of the disease pathology. Still, to our knowledge, there are as yet no* in vivo* PET studies of A_2A_ receptor expression in AD or in animal models of AD.

## 4. A_2A_ Receptors in Multiple Sclerosis

### 4.1. Pathological Characteristics of Progressive Multiple Sclerosis

MS is traditionally considered an autoimmune disease, where an immune attack towards myelin leads to demyelination and bouts of neurological symptoms [96]. Neuropathological studies have demonstrated that, in addition to the active focal inflammation, there is also an ongoing neurodegenerative process, which starts already early on in the relapsing remitting multiple sclerosis (RRMS) phase of the disease, in both the gray matter and the white matter, and leads to gradual axonal damage, neuronal loss, and CNS atrophy [97]. With time, the RRMS disease advances to a secondary progressive phase (SPMS), with an alteration in neuropathological findings [98]. In addition to the focal inflammatory lesions, increased spreading of the inflammatory process into the so-called normal appearing white matter (NAWM) with involvement of brain resident glial cells is seen [98]. This inflammation can be measured* in vivo* using translocator protein-18 kDa (TSPO) PET imaging [99, 100]. The widespread microglial activation presumably contributes to the ongoing neurodegenerative process leading to clinical disease progression, but in general the mechanisms of neurodegeneration in progressive MS are presently relatively poorly understood. Importantly, better understanding and better alternatives for* in vivo* measurement of the pathological processes leading to disease progression would enhance therapeutic development for this undertreated condition [101].

### 4.2. Evidence of the Role of A_2A_ Receptors in Multiple Sclerosis Pathogenesis

**Figure 2:**
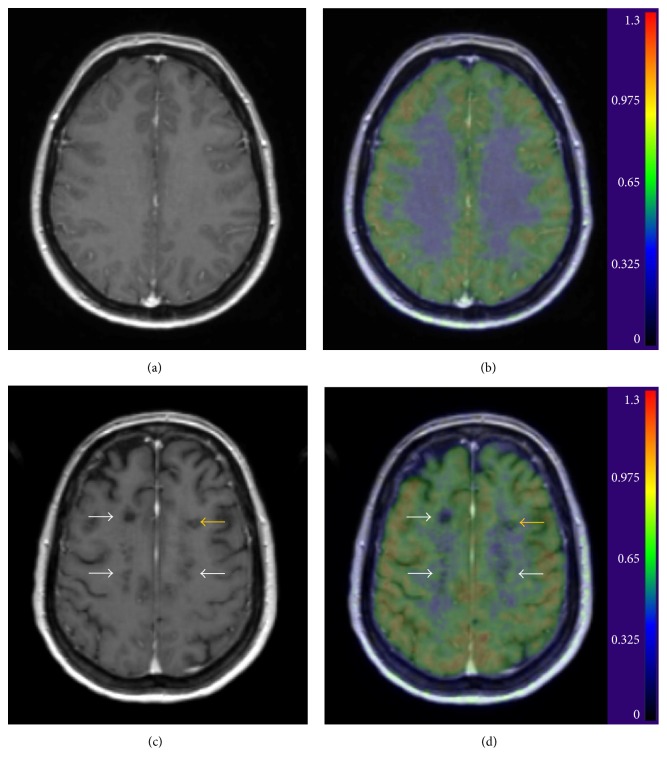
Brain MRI and [^11^C]TMSX PET images from a 45-year-old healthy female (a and b, resp.) and of a 48-year-old female with SPMS (disease duration: 6 years, EDSS 7.5) (c and d, resp.). The images represent axial views from gadolinium-enhanced T1 images (a and c) and parametric [^11^C]TMSX PET images with each voxel's intensity representing the distribution volume (*V*_*T*_, ml/cm3) value of the ligand fused with the T1 image (b and d). A pattern of increased [^11^C]TMSX binding can be observed around the T1 hypointense lesions (white arrows) and within the mildly active plaque in the frontal white matter (yellow arrow) of the SPMS patient compared to the lower, homogeneous binding in the white matter of the healthy control. Figure reprinted with permission from Rissanen et al. (2013) [13].

Direct data on the role of A_2A_ receptors in MS is still scarce, but* in vivo* PET imaging studies using the A_2A_ receptor-binding radioligand [^11^C]TMSX have demonstrated that A_2A_ receptor expression is increased in the NAWM of patients with SPMS compared to age- and sex-matched controls (Figure 2) [13]. Importantly, increased binding in the NAWM correlated with increased clinical disability score (EDSS) and decreased fractional anisotropy (FA) in diffusion tensor imaging (DTI) of SPMS patients, suggesting that the A_2A_ receptors have a likely role in the disease pathogenesis. In respective areas of normal appearing MS brain, increased microglial activation has been demonstrated using TSPO-binding radioligand [^11^C]PK11195 and PET [99]. The identity of A_2A_ receptor-expressing cells in the context of MS is yet to be confirmed. It is nevertheless plausible to hypothesize that activated glia could be among the cell types expressing A_2A_ receptor in the SPMS NAWM, as A_2A_ receptor expression on activated glia has been demonstrated in other settings involving an inflammatory or neurodegenerative environment [63, 65, 102]. Interestingly, increased adenosine levels have been demonstrated in the cerebrospinal fluid and serum of MS patients compared to controls [103, 104]. Moreover, high consumption of coffee (caffeine is a nonspecific antagonist of A_1_ and A_2A_ receptors) associates with decreased susceptibility risk of MS [105] and with reduced risk of progression of RRMS [106], also suggesting that A_2A_ receptor signaling might have a role in evolvement of MS. No clinical trials targeting A_2A_ receptors in MS have been performed, but EAE studies suggest that adenosine signaling might have a robust effect on CNS inflammation, as discussed below.

### 4.3. Evidence of the Role of A_2A_ Receptors in EAE

Treatment of EAE with A_2A_ receptor antagonists such as caffeine or SCH58261 has been shown to significantly reduce clinical scores in multiple mice and rat models of EAE [57, 58, 107–109]. Accordingly, infiltration of inflammatory cells is decreased in the cerebral cortex and spinal cord [58, 107, 109], and demyelination is reduced in these animals [107, 109]. Moreover, mice deficient in CD73 molecule, an ectoenzyme that catalyzes ATP into adenosine, have significantly milder EAE disease and little immune cell infiltration [58]. This supports the notion that preventing stimulation of the A_2A_ receptors within the CNS helps ameliorate EAE.

Conversely and surprisingly, other studies show that genetic removal of A_2A_ receptors results in initial worsening of EAE, after which disease score returns to level of wild type controls [57, 102]. Here histopathology accordingly shows initial increased infiltration of CD4+ T lymphocytes and increased reactivity of microglial activation markers CD11b+/F480+ and Iba-1 in the brain and spinal cord [57, 110]. Interestingly, treatment with A_2A_ receptor agonists from time of immunization (day 0) reduces EAE scores [102, 111], but delayed treatment causes an opposite effect and exacerbates the disease. The opposite is seen with A_2A_ receptor antagonists: treatment with caffeine from day 0 leads to higher mean EAE scores and treatment from day 10 results in lower mean EAE scores [109].

## 5. A_2A_ Receptors in Ischemia and Stroke

Adenosine is excessively released from cells under ischemic conditions [3]. A_2A_ receptor expression in rat brain is increased in the striatum on neurons and microglia following cerebral ischemia [112]. A_2A_ receptors can be beneficially targeted under ischemic conditions, as A_2A_ receptor blockade by genetic deletion of the receptor or pharmacological inhibition protects against cerebral ischemia and ischemia-reperfusion injury in multiple animal studies [113–123]. The protective effect is possibly due to inhibition of glutamate outflow [30, 119]. Because global deletion of A_2A_ receptors seemed protective against ischemia, Yu et al. [124] tested the effect of selective deletion of A_2A_ receptors from bone marrow-derived cells (BMDC) and found that selective reconstitution of A_2A_ receptors on BMDCs reinstated the ischemic brain injury in global A_2A_ receptor knockout mice. Accordingly, selective lack of A_2A_ receptors in the BMDC compartment was sufficient to abolish the protective effect of A_2A_ receptor genetic deletion.

Although the literature on the beneficial effect of the A_2A_ receptor antagonists in ischemia is abundant, some studies suggest that the protective effect of the receptor blockade is lost following excessive reperfusion injury. A recent study suggests that, although A_2A_ receptor antagonists initially protect against transient ischemic injury, the protective effect is lost 7 days after ischemia despite chronic treatment with the antagonist (twice a day) [125]. Similarly, chronic 8-(3-chlorostyryl) caffeine treatment (s.c.) did not show any effect on infarct volume at 72 hours after permanent occlusion of the middle cerebral artery (MCAo) [126] and genetic deletion even worsened ischemic injury in young mice when assessed at 5 days after permanent occlusion of the common carotid artery [127]. Interestingly, A_2A_ receptor agonist CGS21680 (i.p.) was shown to reduce infarct volume (rat cortex but not striatum), microglial activation, and granulocyte infiltration into the brain following transient MCAo when assessed 7 days after ischemia [128].

## 6. A_2A_ Receptor-Binding Radioligands in Human PET Studies

PET imaging of A_2A_ receptors has been used in clinical research in humans but is not generally available or utilized in routine clinical practice. In the clinical diagnostics of neurodegenerative diseases, [^123^I]*β*-CIT-SPECT (single-photon emission computed tomography) can be used for imaging dopamine transporter availability for differential diagnostics of early or atypical PD, [^11^C]PIB for identifying amyloid pathology in early AD if routine morphological imaging is normal, and [^18^F]FDG (2-deoxy-2-[fluorine-18]fluoro-D-glucose) for detecting hypometabolism and differentiating dementia with Lewy bodies (DLB) or frontotemporal lobe degeneration (FTD) from AD. In addition, [^123^I]*β*-CIT-SPECT may aid in differentiating between DLB and AD. For imaging neuroinflammation, [^18^F]FDG could theoretically be used for detecting hypermetabolism, but due to its unspecificity, it is of limited value in clinical practice compared to routine MRI imaging and cerebrospinal fluid (CSF) analyses. Thus, when imaging the detailed mechanisms of A_2A_ receptors in neuroinflammation, more specific probes, such as A_2A_ receptor-binding radioligands, are needed.

**Figure 3:**
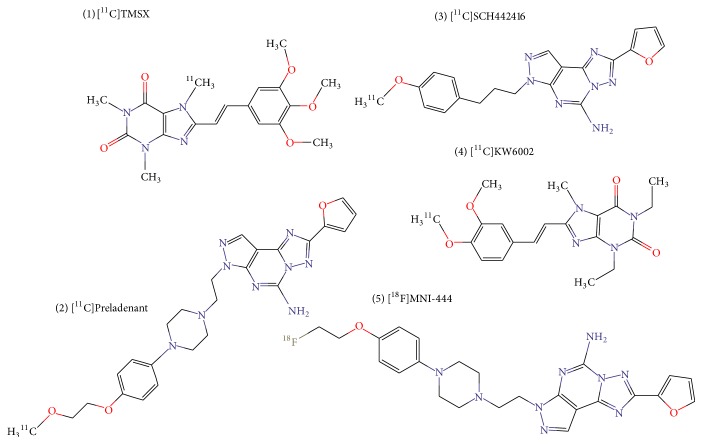
Chemical structures of A_2A_ receptor-binding radioligands. ((1) [^11^C]TMSX, (2) [^11^C]Preladenant, (3) [^11^C]SCH442416, (4) [^11^C]KW6002, (5) [^18^F]MNI-444).

In the healthy CNS, human* in vivo* PET studies demonstrate greatest A_2A_ receptor ligand binding in the basal ganglia, whereas low radiotracer accumulation was shown in cortical areas and cerebellum [19, 129–131]. Subject age does not seem to affect striatal A_2A_ receptor radioligand binding [132]. Regarding evaluation of disease-related A_2A_ receptor expression* in vivo*, interest in PD therapy development has clearly been the driving force. Here, the main focus has been the variation in the A_2A_ receptor level within the striatum, according to disease stage and medication, as discussed above [15, 16, 133]. Several ligands for imaging the A_2A_ receptors have been developed and five of them, that is, [^11^C]TMSX, [^11^C]Preladenant, [^11^C]SCH442416, [^18^F]MNI-444, and [^11^C]KW6002, have been tested in human subjects. Their chemical structures are presented in Figure 3. Below, we discuss the characteristics and the usability of these five radioligands.

### 6.1. [^11^C]TMSX

[^11^C]TMSX is a methylxanthine analog of KF17387. It is the most widely used A_2A_ receptor radioligand and its binding to A_2A_ receptors in humans has been described in the brain [134], myocardium [135, 136], and skeletal muscle [137, 138]. [^11^C]TMSX (previously named KF18446) was first developed by Ishiwata et al. [139] in search of more 2A receptor selective ligands after previously tested xanthine-type ligands had proven poor A_2A_ selectivity over A_1_ and high nonspecific binding [140]. In the rat, [^11^C]TMSX shows relatively low affinity for the A_2A_ receptor (Table 1) and about 270-fold selectivity to A_2A_ receptors over A_1_ [139]. In human brain, A_2A_ receptor antagonist theophylline reduced [^11^C]TMSX binding in the putamen by 4.5% and in the nucleus caudatus by 8%, but not in other areas outside of striatum [134]. Specific binding is highest in the striatum, with reported binding potential (BP) of 1.2–1.25 in the putamen [19] and DVR 1.67 in the striatum [141], followed by lower binding in the thalamus, cerebellum, brainstem, and the cortex [19]. Both the centrum semiovale [142] and the cerebral cortex [132] have been used as reference for calculating TMSX binding. In addition, a semiautomated method using supervised clustering for the extraction of gray matter reference region has been developed [141].

Acquiring metabolite corrected plasma input function via arterial cannulation and repeated arterial sampling for the measurement of the radioligand activity and metabolism is considered the golden standard in brain PET image analyses especially with novel ligands without a priori knowledge of the ligand's kinetics and metabolism. However, this methodology can be unpleasant for the study subjects, may be prone to errors, and requires more expert personnel. Therefore, optional methods for obtaining plasma input function have been developed, including independent component analysis [142] and intersectional searching algorithm with averaging and clustering of PET data (robust EPISA) [150]. Importantly, plasma input methods can be affected by the fraction of radioactive metabolites. Using nonmetabolite corrected input has been reported to underestimate the [^11^C]TMSX distribution volume (*V*_*T*_) by approximately 5% when compared with metabolite corrected plasma input [142]. Consequently, a noninvasive, validated method for obtaining metabolite corrected population-based plasma input function for [^11^C]TMSX has been developed and validated [141]. Dosing and blood sampling under dimmed light is required due to [^11^C]TMSX photoisomerization.

### 6.2. [^11^C]SCH442416

[^11^C]SCH442416 (5-amino-7-(3-(4-[^11^C]methoxy)phenylpropyl)-2-(2-furyl)pyrazolo[4,3-e]-1,2,4-triazolo[1,5-c]pyrimidine) was the first suitable nonxanthine radioligand for the imaging of A_2A_ receptors. In a blocking study with vipadenant (an A_2A_ receptor antagonist), the highest radioligand binding measured as metabolite corrected* V*_*T*_ was seen in putamen (*V*_*T*_ ~ 0.6 ml/cm^3^), followed by caudate, nucleus accumbens, thalamus, and cerebellum (*V*_*T*_ ~ 0.3 ml/cm^3^) [149]. A_2A_ receptor blocking with vipadenant resulted in notable 3-4-fold reduction in total [^11^C]SCH442416 binding (*V*_*T*_) in striatal ROIs and also in about up to 2-fold reduction in cerebellum. Two later studies have shown very different specific binding potentials in the putamen when using the cerebellum as a reference region for the estimation of specific radioligand binding. Grachev et al. [148] reported the average binding potential (BP_ND_) of five healthy subjects in the putamen as 2.47 ± 0.84, whereas Ramlackhansingh et al. [15] reported the average BP_ND_ of six healthy controls (control group in a PD study) to be as low as 0.99 ± 0.21. The intersubject variability was, however, fairly large in the aforementioned study (BP_ND_ 1.12–3.82 in the putamen) [148]. In both studies spectral analysis with metabolite corrected arterial plasma input and cerebellum as a reference region were used for the quantification of specific radiotracer binding. Whether or not the region of interest (ROI) for cerebellum as reference region was defined in a similar manner in both studies—a possible source of discrepancy in the results—is not known. Finally, neither study reported the use of coffee or other caffeine-rich beverages prior to imaging session. In PET imaging studies using other A_2A_ ligands [13, 132], abstinence from caffeinated drinks has been required at least for 12 hours before the scan in order to rule out the possible blocking effect by caffeine.

### 6.3. [^11^C]Preladenant

[^11^C]Preladenant has high affinity for the A_2A_ receptor and >1000-fold selectivity to the A_2A_ over the other adenosine receptor subtypes [143]. First human study with [^11^C]Preladenant was recently published [129]. Here, eight healthy male subjects were tested. Approximately 78% of Preladenant was unmetabolized at 60 minutes. In a rat study, 17% of the total radioactivity in the brain was due to radioactive metabolites at 60 minutes [151]. It will be thus necessary to take these radiometabolites into consideration in the kinetic modeling, with metabolite corrected input function. [^11^C]Preladenant has a DVR of 7.9 ± 2.3 in the putamen and shows lower binding in the frontal cortex, thalamus, and cerebellum [129]. In rhesus monkeys, pretreatment with Preladenant before PET imaging with [^11^C]Preladenant reduced striatal binding to extrastriatal levels but also reduced extrastriatal binding [144]. Cerebellum was nevertheless used as a reference region.

### 6.4. [^18^F]MNI-444

[^18^F]MNI-444 is the only [^18^F]-labeled A_2A_ radioligand used in humans. It has relatively high affinity (*K*_*i*_ = 2.8 nM) for the human recombinant A_2A_ receptor [145]. Reported BP_ND_ to putamen is 4.7 ± 0.63, to globus pallidus 3.67 ± 0.69, and to caudate 2.69 ± 0.74 [130]. Also in these studies, cerebellum was used as a reference region although a dose-independent reduction in cerebellar binding was found in preblocking with Tozadenant and Preladenant in the rhesus monkey [146].

### 6.5. [^11^C]KW6002

In rodent and human studies, [^11^C]KW6002 shows high binding in the striatum, but binding is also detected in the cerebellum and thalamus. In addition, preblocking with A_2A_ receptor antagonist KW6002 reduced [^11^C]KW6002 binding to A_2A_ receptors in all studied brain regions [152, 153]. The authors concluded that the extrastriatal binding could be explained by binding to A_1_ and A_2B_ receptors, although no effect of A_2B_ receptor antagonist on [^11^C]KW6002 binding was found [153]. Due to its inadequate specificity, this ligand has not been further developed.

### 6.6. Challenges in A_2A_ Receptor PET Imaging

Even though the highest specific [^11^C]TMSX binding occurs in putamen and caudate, there appears to be some specific, albeit lower, A_2A_ receptor binding in extrastriatal tissues such as cortical gray matter and cerebellum. The rate of specific binding, calculated as BP/*V*_*T*_, has been reported to be as high as 53% in cerebellum and 37.8–42.7% in cerebral cortex for [^11^C]TMSX [19]. Similarly, the previously mentioned blocking studies with newer A_2A_ receptor radioligands demonstrate the presence of some specific A_2A_ receptor binding in extrastriatal gray matter. Therefore, both cerebellum and cerebral cortex appear as less than optimal reference regions. Moreover, in diseases with widely spread pathology, such as MS, a common, anatomically defined reference region that is presumably free of disease pathology, inflammatory activity, and possible specific binding is difficult to find. Also, when studying diseases with predominant white matter affliction, such as MS, centrum semiovale is not a feasible reference region either, even though in healthy controls the A_2A_ receptor binding in central white matter is negligible. In order to overcome these issues, a method for supervised clustering of the reference region has been developed and validated for [^11^C]TMSX based on the same algorithm used for [^11^C]PK11195 studies (SuperPK software) [154]. Importantly, this method is based on predefined kinetic classes, where the shape of the time activity curve (TAC) in the gray matter reference region is considered to represent nonspecific binding as opposed to the high specific binding with different TAC shape [141].

## 7. Conclusion

There is increasing interest in the therapeutic development of A_2A_ receptor antagonists and agonists in a variety of neurological conditions. A_2A_ receptors are ubiquitously expressed in various areas of the CNS, but their significance in the context of the different CNS diseases still needs clarification. Pathological processes in CNS diseases are particularly difficult to investigate for reasons such as the difficulties in obtaining representative biopsies from the brain. PET imaging, on the other hand, provides an excellent opportunity to evaluate disease-specific pathology* in vivo, *by allowing quantitative study of the receptors of interest in an appropriate pathological environment in situ. With the increasing variety of A_2A_ receptor-binding PET ligands available for use in human* in vivo *PET imaging, there is good likelihood that PET imaging will improve our understanding of the involvement of A_2A_ receptors in the pathophysiology and pathogenesis of brain diseases, both in the neuronal compartment of the basal ganglia and in relation to inflammation, such as in progressive MS. Groups of patients can be studied cross sectionally at various stages of a given disease or, alternatively, PET imaging can be applied longitudinally to evaluate alterations in the A_2A_ receptor in the course of the disease or in response to treatment. PET imaging of neuroinflammation has relied heavily on TSPO-binding radioligands, but methodological challenges related to TSPO-imaging has directed the field to actively seek alternative imaging probes. A_2A_ receptor PET imaging provides one such alternative that is worth further exploring.

## Figures and Tables

**Table 1:** Currently available A_2A_ receptor binding radioligands.

	Affinity to A_2A_ (nM)/selectivity over other adenosine receptors	Characteristics of specific binding	*V*_*T*(putamen)_ with metabolite corrected arterial plasma input	A_2A_ receptor occupancy/blocking studies
[_ _^11^C]TMSX	*K*_*i*(rat)_ = 5.9/selectivity over A_1_: 270-fold [139]	BP in anterior and posterior putamen: 1.25 ± 0.17 and 1.20 ± 0.16, respectively (centrum semiovale as reference region) [19] DVR in striatum (clustered gray matter as reference region) 1.674 [141]	1.72 [134] 1.66–1.69 [19] 1.11 (striatum) [13]	Theophylline infusion reduced *V*_*T*_ in putamen by 4.5% and in the nucleus caudatus by 8%. No effect on other areas [134].

[_ _^11^C]Preladenant	*K*_*i*(human)_ = 1.1 *K*_*i*(rat)_ = 2.5/selectivity over (human) A_1_, A_2B_, A_3_: >1000-fold [143]	DVR in putamen 7.9 ± 2.3 (2 tissue model) 7.7 ± 1.9 (LGRM) (cerebellum as reference region) [129]	4.5 ± 1.3 [129]	Preladenant pretreatment reduced striatal *V*_*T*_ to level of extrastriatal binding in rhesus monkeys [144].

[_ _^18^F]MNI-444	*K*_*i*(human recombinant)_ = 2.8 [145]	BP 4.7 ± 0.63 (cerebellum as reference region) [130]	3.26 ± 0.98 (with LGA) [130]	Preblocking with Tozadenant or Preladenant reduced total binding (SUV) to the level of extrastriatal (cerebellum) binding at the highest dose in rhesus monkeys. Also <15% reduction in cerebellar *V*_*T*_ with preblocking was observed [146].

[_ _^11^C]SCH442416	*K*_*i*(human)_ = 0.048/selectivity over A_1_, A_2B_, A_3_ >20000-fold [147]	BP 0.99 ± 0.21 (cerebellum as reference region) [15] BP_ND_ 2.47 ± 0.84 (cerebellum as reference region) [148]	≈0.6 [149]	Preblocking with Preladenant led to dose-dependent A_2A_ receptor occupancy in the striatum (at 200 mg 88–105%), with corresponding decrease in [_ _^11^C]SCH442416 binding (BP_ND_) [148]. Preblocking with vipadenant led to approximately 3-4-fold reduction [_ _^11^C]SCH442416 V_T_ in caudate and putamen and also to an up to 2-fold reduction in cerebellum. Dose-dependent receptor occupancy observed in putamen, caudate, nucleus accumbens, and cerebellum (on average from 74% to 95% with 2.5–100 mg dose), but not in thalamus [149].
